# Pediatric Conjunctivitis: A Review of Clinical Manifestations, Diagnosis, and Management

**DOI:** 10.3390/children10050808

**Published:** 2023-04-29

**Authors:** Matthew J. Mahoney, Ruegba Bekibele, Sydney L. Notermann, Thomas G. Reuter, Emily C. Borman-Shoap

**Affiliations:** Department of Pediatrics, University of Minnesota, Minneapolis, MN 55454, USA

**Keywords:** pediatric conjunctivitis, pink eye, pediatric ophthalmology

## Abstract

Conjunctivitis is a common pediatric problem and is broadly divided into infectious and non-infectious etiologies. Bacterial conjunctivitis makes up the majority of cases in children and often presents with purulent discharge and mattering of the eyelids. Treatment is supportive with an individual approach to antibiotic use in uncomplicated cases since it may shorten symptom duration, but is not without risks. Viral conjunctivitis is the other infectious cause and is primarily caused by adenovirus, with a burning, gritty feeling and watery discharge. Treatment is supportive. Allergic conjunctivitis is largely seasonal and presents with bilateral itching and watery discharge. Treatment can include topical lubricants, topical antihistamine agents, or systemic antihistamines. Other causes of conjunctivitis include foreign bodies and non-allergic environmental causes. Contact lens wearers should always be treated for bacterial conjunctivitis and referred to evaluate for corneal ulcers. Neonatal conjunctivitis requires special care with unique pathogens and considerations. This review covers essential information for the primary care pediatric provider as they assess cases of conjunctivitis.

## 1. Introduction

Conjunctivitis, commonly called “pink eye”, refers to inflammation or infection of the conjunctiva. The conjunctiva is the thin mucous membrane that lines the inside of the eyelids and the surface of the globe up to the limbus, where the sclera and cornea meet. It is divided into the following two portions: the bulbar portion, covering the globe, and the tarsal portion, covering the lids. It is usually transparent; however, it can become injected and pink or red when inflamed, leading to the colloquial term “pink eye”. Conjunctivitis can vary in severity, ranging from mild redness associated with tearing to subconjunctival hemorrhage with purulent discharge and edema of the conjunctiva or eyelid.

The classification of pediatric conjunctivitis is typically by etiology, broadly categorized into infectious and non-infectious causes. Most cases of pediatric conjunctivitis are infectious, either bacterial or viral. Non-infectious conjunctivitis includes allergic conjunctivitis as well as conjunctivitis due to foreign bodies, environmental causes, or contact lens overwear. There are certainly other, more serious, causes of pink eye such as cellulitis, uveitis, endophthalmitis, and acute glaucoma, which may have a similar presentation; however, those causes are beyond the scope of this discussion.

The majority of cases of pediatric conjunctivitis are managed by primary care providers rather than eye-specific providers [1]. There are no widely accepted guidelines for the management of conjunctivitis in children and widely varying practices from clinician to clinician have been noted [2]. The goal of this literature review is to summarize the current evidence on the clinical manifestations, diagnosis, and management of conjunctivitis for primary care providers.

## 2. Making the Diagnosis

When a child presents with “pink eye”, the evaluation should first start with a review of the history, signs, and symptoms to determine the etiology. It is essential to obtain information such as the length of symptoms, whether one or both eyes are affected, and a description of drainage from the eye(s), if any. Associated symptoms may also give a clue for the etiology, particularly if there are co-occurring viral symptoms such as a cough, sore throat, fever, or rash. If the patient has had trauma to the eye or if their symptoms have a predominance for certain times of the year, this may also provide guidance toward understanding the etiology. Depending on the age of the child, it may be appropriate to ask if they have noticed any changes to their vision or if a foreign body sensation is present.

The physical examination should always begin with a vision measurement, testing each eye separately with a Snellen chart. For children too young to participate with a Snellen chart test, near vision can be broadly measured by seeing if patients can focus on a book, toy, or their caregiver. If visual acuity appears to be affected, a referral should be made to a pediatric ophthalmologist for further evaluation.

Physical examination should continue with the use of a penlight. When examining the pupils and anterior segment, attention should be given to the size of the pupil and if it is reactive to light. If there is discharge present, it is important to note the consistency, color, and amount of it. In addition, the conjunctiva should be examined to determine if the entire conjunctiva is affected or if there is a specific area that is more erythematous. For clinicians that are comfortable with it, inverting the eyelid can also provide clues to the etiology. A fundoscopic exam is not typically useful in differentiating between the various etiologies. Laboratory testing and imaging are also not typically necessary for cases of uncomplicated conjunctivitis.

A thorough history and physical exam can give clues to the etiology and management of “pink eye,” as seen in Table 1 and Table 2, respectively. However, it is important to note that clinical presentation is often non-specific. While significant research has been devoted to predicting the causative agent based on symptoms, few studies have demonstrated the ability to successfully achieve this. A 2003 meta-analysis did not find any evidence for the diagnostic usefulness of clinical signs and symptoms to differentiate bacterial from viral conjunctivitis [3]. However, a more recent meta-analysis from 2022 found that bacterial conjunctivitis may in fact be the more common cause of conjunctivitis in children [4], with as many as 70% of conjunctivitis cases in children. The same meta-analysis found that adults presenting with acute conjunctivitis had an identified bacterial etiology much less often, only about 16% of the time.

A multi-center study of adults with bacterial conjunctivitis demonstrated that the symptoms can widely vary. Of those patients with positive bacterial cultures, 65% had burning, 58% had itching, and 35% had serous or no discharge at all [5]. Associated symptoms may give clinical clues to the bacterial, viral, or other causes of conjunctivitis. For example, the presence of mucopurulent discharge or otitis media is suggestive of a bacterial etiology. Concomitant pharyngitis, pre-auricular lymphadenopathy, and known contacts with red eye all suggest viral etiologies [4].

### 2.1. Bacterial Conjunctivitis

Acute bacterial conjunctivitis is common in children, with more than 50% of conjunctivitis cases being bacterial in origin [6,7]. Common pathogens include *Haemophilus influenzae*, *Streptococcus pneumoniae*, and *Moraxella catarrhalis*. Of those, *H. influenzae*, most commonly non-typeable *H. influenzae*, remains the most common cause of bacterial conjunctivitis, present in up to 70% of cases [8]. Presenting symptoms are commonly eye redness and significant mucopurulent discharge, frequently yellow to green in color. Patients with acute bacterial conjunctivitis often complain of eyes that are mattered and adhered in the morning [3]. On examination, patients typically have mucopurulent discharge at the lid margins that reappears quickly after wiping the lids, usually within minutes. 

### 2.2. Viral Conjunctivitis

Viral conjunctivitis makes up a significant portion of acute conjunctivitis cases, and the vast majority are caused by adenoviruses. Presentation often consists of a burning or gritty feeling with watery discharge. This typically has an abrupt onset, starts with one eye, and infects the other eye within 24 to 48 h. This can be accompanied by a viral prodrome with fever, lymphadenopathy (particularly preauricular), pharyngitis, and/or upper respiratory tract infection. A clinical exam typically demonstrates prominent conjunctival injection with watery discharge and a follicular appearance of the tarsal conjunctiva.

While adenoviruses cause most of these cases, other viral causes must be considered. Herpes simplex virus (HSV) can present in a similar manner to adenoviral conjunctivitis with watery eye discharge, often co-occurring with characteristic HSV vesicular eruptions on the face [9]. Fluorescein examination will reveal multiple small branching epithelial dendrites on the surface of the cornea [10]. Molluscum contagiosum, a poxvirus known for causing skin colored, umbilicated papules, can present with lesions on the eyelid and lead to follicular conjunctivitis [11]. Picornaviruses such as Enterovirus 70 and Coxsackievirus A24 can cause acute hemorrhagic conjunctivitis and have led to multiple pandemics, particularly in developing countries [12].

Coronaviruses have not been known to cause ocular manifestations in the past; however, the novel coronavirus disease 2019 (COVID-19) led to viral conjunctivitis. A meta-analysis demonstrated that approximately 1 in 10 cases of COVID-19 had ocular involvement, primarily characterized by dry eye, redness, and tearing [13]. In pediatric patients specifically, conjunctivitis is the most common ocular manifestation of COVID-19. A study of 15 neonates with COVID-19 showed that more than 70% had chemosis (conjunctival edema) and hemorrhagic conjunctivitis [14]. Conjunctivitis appears to be more likely associated with a systemic inflammatory reaction rather than a direct viral infection [13,14]. More serious complications, such as orbital cellulitis, retinal vein occlusion, and optic nerve abnormalities, have been associated with COVID-19; however, these are more rare [13].

### 2.3. Allergic Conjunctivitis

Allergic conjunctivitis is a type I hypersensitivity reaction, most commonly to airborne allergens such as pollen, dander, dust, or molds. This IgE-mediated reaction causes mast cell degranulation, leading to the release of histamine and other pro-inflammatory mediators. This occurs in approximately 1 in 5 children, with a peak age from late childhood to adolescence [15]. In fact, allergic conjunctivitis is the most common ocular complaint to the pediatric healthcare provider [16]. Its presentation is typically bilateral with watery discharge, chemosis, and crusting on lid margins in the morning. It tends to occur when allergen levels, such as pollen, are at their peak. The key differentiating factor is itching, sometimes occurring with other atopic symptoms such as nasal congestion, cough, or sneezing. The clinical exam findings are similar to viral conjunctivitis with watery discharge and a follicular appearance of the tarsal conjunctiva. A clinical algorithm from a 2017 review laid out helpful features to help rule out allergic conjunctivitis [17]. For example, photophobia, eye pain, and blurry vision are not expected with allergic conjunctivitis and should prompt referral to an eye specialist. Conditions that can serve as mimickers of allergic conjunctivitis include blepharitis and meibomian gland dysfunction [18]. 

### 2.4. Foreign Body

Foreign bodies in the eye can present with eye pain, foreign body sensation, and sensitivity to light. These injuries occur in approximately 2 per 1000 in the US and make up 31% of eye-related diagnoses during ER visits [19]. Ocular foreign bodies are particularly prevalent in the setting of recent eye trauma [20]. Nearly half of eye foreign bodies in pediatric patients are due to wood, sand, and dust and high velocity injuries from BB, paintball, and airsoft guns are especially common in adolescent males [21]. Even without a history of trauma, a physical examination of the eyes is vital in both assessing the acuity of the injury and visualizing any potential foreign bodies. The physical exam should include visual acuity, full eyelid evaluation, and pupillary exam; however, if symptoms such as sudden eye pain or vision loss cause concern for globe rupture, applanation tonometry and scleral depression are not recommended. Slit lamp evaluation with fluorescein staining can be used to visualize corneal abrasions caused by foreign objects [20].

**Table 1:** Clinical Symptoms and Physical Exam Findings of Various Etiologies of Conjunctivitis.

Conjunctivitis	Clinical Symptoms	Physical Exam
Bacterial	Acute bilateral or unilateral purulent discharge with mattering and adherence	Significant discharge, typically green or yellow
Viral	Acute burning or gritty feeling often accompanied by prodromal symptoms, such as fever, cough, and rhinorrhea	Watery discharge and follicular appearance to conjunctiva
Allergic	Bilateral eye itchiness often accompanied by atopic symptoms, typically chronic and seasonal	Watery discharge, chemosis, and follicular appearance to conjunctiva
Foreign Body	Acute eye pain with foreign body sensation and sensitivity to light	Foreign body visualized and/or corneal abrasion visualized with fluorescein staining

### 2.5. Contact Lens Overwear

Conjunctivitis in a patient who wears contact lens always necessitates further investigation. Common complications from contact lens wear include discomfort and dry eyes, which may lead to red, irritated eyes, oftentimes secondary to corneal hypoxia. Overwear of lenses can result in giant papillary conjunctivitis, an inflammation caused by multiple factors including mechanical rubbing from the contact lens on the upper eyelid, corneal hypoxia, and cellular mitosis. Two serious complications that can result from contact lens use include corneal ischemia leading to neovascularization and infectious keratitis, which can lead to a corneal ulcer [22]. Examination, particularly by an eye-specific provider, can help differentiate between giant papillary conjunctivitis requiring a lens break or a contact lens-related corneal ischemia or ulcer, which must be treated immediately to avoid vision loss. 

### 2.6. Non-Allergic Environmental Causes

Conjunctivitis that is not infectious or allergic in etiology is broadly defined as unspecific conjunctivitis of unknown origin (UCUO). This commonly presents with eye redness and foreign body sensation. There appears to be a specific link between pollution in the environment and the prevalence of UCUO. A study of 132 children demonstrated a significant increase in the incidence of UCUO compared to the total conjunctivitis cases in residents of areas with higher air pollution [23]. In Taiwan, a study identified that high levels of specific air pollutants, including ozone, nitrogen dioxide, particulate matter, and sulfur dioxide, significantly increased the prevalence of outpatient visits for non-specific conjunctivitis [24]. Particularly in patients who live in settings with higher air pollution, non-allergic environmental causes and UCUO should be considered.

### 2.7. Neonatal Conjunctivitis

Neonatal conjunctivitis, also known as ophthalmia neonatorum, is conjunctivitis that occurs during the first 28 days of life. Similar to conjunctivitis in older children, neonates with bacterial conjunctivitis typically present with purulent discharge, while those with viral conjunctivitis more commonly have watery discharge [25]. Most neonatal conjunctivitis is bacterial in etiology, with *Chlamydia trachomatis* causing the majority of cases (approximately 40% of total cases [26]). Chlamydial conjunctivitis typically presents five days to two weeks after birth with unilateral or bilateral conjunctival redness and watery secretions [27]. This can progress with time to purulent discharge and the formation of pseudomembranes, yellow-white membranes visible on the tarsal conjunctiva [28]. *Neisseria gonorrhoeae* is another cause of bacterial conjunctivitis and results in significant redness and swelling, lid edema, and purulent discharge. This occurs earlier than chlamydial conjunctivitis, typically around two to five days after birth [27]. 

Viruses cause a significantly lower proportion of neonatal conjunctivitis and are primarily caused by the herpes simplex virus (HSV). HSV conjunctivitis in neonates usually occurs from infection in the birth canal and a neonate who presents with conjunctivitis after known exposure to HSV should receive extra attention [29]. On examination, clues for HSV conjunctivitis are similar to those in older children with vesicular eruptions on the face, conjunctival injection, and a hazy cornea secondary to edema [29].

Chemical conjunctivitis typically presents in the first 24 h of life. Silver nitrate drops have been used as prophylaxis against infectious causes of neonatal conjunctivitis; however, they frequently result in irritation to the conjunctiva [26]. Since the removal of silver nitrate from general use in the 1980s, the prevalence of neonatal chemical conjunctivitis has decreased significantly [30]. Today, most causes of neonatal chemical conjunctivitis in the United States are thought to be linked to the use of prophylactic antibiotics such as erythromycin ophthalmic ointment or drops and gentamycin drops [31]. 

Another cause of eye drainage that can mimic neonatal conjunctivitis is congenital nasolacrimal duct obstruction. The nasolacrimal duct starts with the puncta in the eye and carries tears through the lacrimal canaliculi to the nasal cavity. Congenital nasolacrimal duct obstruction can cause a so-called overflow of tears and lead to either a watery or purulent discharge, depending on whether the proximal or distal portion of the duct is affected [32]. It is a common presentation and is present in around 20% of infants, with 95% of those affected showing symptoms in the neonatal period [25].

## 3. Treatment

### 3.1. Bacterial Conjunctivitis

The vast majority of cases of bacterial conjunctivitis are self-limiting, lasting 7 to 10 days without treatment. While antibiotics have been shown to decrease the duration of symptoms, no differences in sight threatening outcomes have been observed between treatment and non-treatment groups. In a meta-analysis consisting of 11 randomized clinical trials and 3673 patients, there was a 10% increase in the rate of clinical improvement for patients who received early antibiotic treatment compared with the placebo group [33]. A recent study from Finland supported this, showing a more rapid clinical cure in patients treated with antibiotic eye drops, from a mean of 4.0 days with a placebo to a mean of 3.8 days with moxifloxacin treatment [34]. Antibiotics are not without risk, with adverse drug reactions reported by 8% of patients using ophthalmic antibiotics [35]. In addition, studies have shown acquired resistance to pathogenic bacteria in the conjunctiva of children prescribed antibiotics [6,36]. While antibiotics are not required for all cases, contact lens wearers should always be treated with antibiotics due to the increased risk of infection with gram negative organisms and subsequent keratitis.

With that in mind, no treatment, a delayed treatment approach, and immediate treatment all are appropriate responses to suspected uncomplicated bacterial conjunctivitis [37]. A study of 20 clinicians in Colorado demonstrated that the main drivers behind choosing to prescribe or withhold antibiotics were the patient’s clinical presentation, family expectations, antibiotic stewardship concerns, diagnostic uncertainty, and daycare and school policies. They noted that the most critical features to help clinicians differentiate between viral and bacterial conjunctivitis were the association with other upper respiratory symptoms and laterality [2].

Overprescribing of antibiotics is common, particularly in cases where the etiology is uncertain. The COVID-19 pandemic worsened this, likely due to the increase in children being treated over the telephone or virtually without being seen directly by a physician [38]. In many cases, families will assert that treatment with antibiotics is necessary for their child to return to school or daycare [2]. School specific policies widely vary from state to state, but the American Academy of Pediatrics specifically notes that antibiotics should not be required for return to care [2,39]. If treatment is desired, initial treatment would begin with erythromycin ointment or trimethoprim-polymyxin B ophthalmic drops. Symptoms would be expected to improve within one to two days.

### 3.2. Viral Conjunctivitis

Treatment of viral conjunctivitis, including COVID-19 conjunctivitis, is primarily symptomatic through the use of cool compresses and lubricating artificial tears. Adenoviral conjunctivitis is generally self-limited and highly contagious. A study of 56 adults with adenoviral conjunctivitis who were treated in clinics with a single drop of 5% povidone iodine demonstrated reduced viral load and a more rapid improvement in symptoms [40]. While not standard practice at this time, this is likely to be an emerging therapy if larger studies in the future can demonstrate similar symptomatic improvement. Patients should be educated on the ways to prevent the spread of viral conjunctivitis, such as avoiding shared towels or bed linens and washing their hands frequently. In fact, a study of 26 adults with conjunctivitis showed that 46% had positive adenovirus cultures grown from swabs of their hands [41]. Patients should be encouraged to make every attempt to minimize contact with others for 10 to 14 days from symptom onset [37].

### 3.3. Allergic Conjunctivitis

Treatment of allergic conjunctivitis consists of minimizing exposure to the allergen and controlling symptoms. Topical lubricants such as artificial tears or saline can be used to physically wash out the offending allergens. Mild allergic conjunctivitis can be treated with topical antihistamine agents, preferably second generation topical H1-receptor antagonists [42]. If persistent, ophthalmic drops that have both antihistamine activity and mast cell stabilizing properties, such as azelastine or olopatadine, can be used. A step-wise approach may be helpful, starting with topical lubricant, then antihistamines, and finally topical steroids [17]. Of note, topical steroids should only be used in a time-limited fashion, limited to 7 days or less. Systemic antihistamines are frequently used to reduce histamine release, improving both allergic conjunctivitis and other systemic symptoms.

### 3.4. Foreign Body

Many foreign bodies are superficial and benign, yet cause significant pain. All patients with suspected corneal foreign bodies should receive a complete eye examination. Topical NSAIDs such as ketorolac and oral analgesics have been shown to reduce pain and improve patients’ tolerance of the examination [43]. If a foreign body is identified, removal should be completed as soon as possible, usually within 24 h. If foreign body accessibility is limited, emergent foreign body removal should be completed by an ophthalmologist [44]. Upon removal, topical prophylactic antibiotics should be prescribed to prevent superimposed infection. Even if a foreign body is not identified, individuals wearing contact lenses should receive anti-pseudomonal coverage such as ciprofloxacin or gentamicin. For those without contacts, topical bacitracin or erythromycin have been utilized; however, the efficacy of prophylactic antibiotics is still uncertain.

**Table 2:** Treatments of Various Etiologies of Conjunctivitis.

Conjunctivitis	Treatment
Bacterial	Self-limiting within 7–10 days; however, consider erythromycin ointment or trimethoprim-polymyxin B drops
Viral	Symptomatic treatment with cool compresses and artificial tears
Allergic	Avoid allergic exposures and consider use of topical antihistamines
Foreign Body	Foreign body removal with saline irrigation, pain relief with topical NSAIDs or oral analgesics, and topical antibiotics

### 3.5. Neonatal Conjunctivitis

In the United States, ocular prophylaxis against neonatal conjunctivitis with 0.5% erythromycin ophthalmic ointment is the common practice. However, there is an ongoing conversation about the necessity of ocular prophylaxis, given that the rates of gonorrhea in pregnant people have decreased steadily since the 1970s [45]. All pregnant people are screened throughout their pregnancy; thus, the majority of neonates are treated prophylactically despite a minimal risk of them developing gonococcal conjunctivitis [31]. The data surrounding the efficacy of erythromycin ointment on gonococcal conjunctivitis are limited and there is concern for *N. gonorrhoeae* developing resistance to erythromycin [31]. Despite this, the United States Preventative Service Task Force (USPSTF) continues to recommend erythromycin prophylaxis for all newborns. In the USPSTF’s most recent reaffirmation statement on ocular prophylaxis, they cite that the rate of gonococcal neonatal conjunctivitis is currently estimated to be 0.4 cases per 100,000 live births per year and without ocular prophylaxis, transmission rates are as high as 30% to 50% [45]. Despite the fact that all pregnant people are screened for *N. gonorrhoeae*, approximately 6.2% of individuals in the United States do not receive prenatal care, thus would have an increased risk of unknowingly transmitting *N. gonorrhoeae* to their newborn [45]. With this in mind, erythromycin ophthalmic ointment remains the standard of care. 

One side effect that is important for clinicians to be aware of is that erythromycin ophthalmic ointment can lead to a form of chemical conjunctivitis in the first 24 h of life [31]. Neonatal chemical conjunctivitis, whether from erythromycin or silver nitrate, is typically self-limited and resolves within two to four days [30]. For other cases of conjunctivitis, the treatment depends on the etiology. For conjunctivitis caused by *C. trachomatis,* treatment is typically erythromycin ophthalmic drops plus oral erythromycin for a total course of two to three weeks [37]. For cases of conjunctivitis caused by *N. gonorrhoeae*, the treatment is a third-generation cephalosporin, such as ceftriaxone, in a single dose. This is started along with normal saline irrigation to the eyes with hopes to remove the mucopurulent discharge typically present. As with most cases, neonates treated for gonococcal conjunctivitis should also be treated for chlamydial conjunctivitis, given the prevalence of co-infection [37].

## 4. When to Refer

Although certain symptoms and clinical exam findings tend to correspond with specific causes of pink eye, it must be emphasized that clinical manifestations are non-specific and that considerable overlap exists in actual clinical practice. When in doubt, primary care providers should not hesitate to make appropriate referrals to ophthalmology. 

The American Academy of Ophthalmology recommends specific symptoms and conditions that should be referred for further evaluation [37]. Symptoms that should prompt further evaluation include moderate to severe pain, vision loss, constant blurred vision, and severe purulent discharge. Photophobia should also prompt further evaluation, particularly if out of proportion to other symptoms or found in cases of allergic conjunctivitis. In addition, referral should be considered for those with symptoms lasting more than 7 to 10 days, recurrent symptoms, or who do not respond to treatment.

Specific cases also require further evaluation. Patients who wear contact lenses should always be treated, encouraged to discontinue lens wear, and referred for a slit lamp exam to rule out a contact lens-related corneal ulcer. Many cases of allergic conjunctivitis can be treated without a referral; however, if vernal conjunctivitis is suspected, patients should be referred, since it can be vision-threatening. 

## 5. Conclusions

Conjunctivitis is a common complaint in the pediatric primary care office. A clinician can use diagnostic clues from the patient’s history and exam to help determine the likely etiology. In general, bacterial conjunctivitis makes up the majority of cases of pediatric conjunctivitis and presents with purulent discharge and mattering of eyes. Viral conjunctivitis leads to a gritty feeling with watery discharge and is often associated with other upper respiratory symptoms. Allergic conjunctivitis is usually bilateral and coincides with seasonal allergen levels. Other causes of conjunctivitis occur in particular groups, including contact lens wearers, those in high pollution environments, and those with symptoms that go beyond conjunctivitis. The treatment of most cases is supportive with topical lubricants; however, antibiotics are indicated on an individual basis for cases of bacterial conjunctivitis, depending on patient and family preference and the clinician’s approach to treatment. Cases that do not resolve as expected should be referred for additional evaluation by an eye-specific provider. 

## Data Availability

Not applicable.
